# Bowel cancer in a 17 year old: what could be the reason?

**DOI:** 10.1186/1897-4287-10-S2-A69

**Published:** 2012-04-12

**Authors:** M Bowman, E Edwards, A Goodwin, J Kirk

**Affiliations:** 1 Familial Cancer Service, Westmead Hospital, Australia

## 

Inherited conditions predisposing to early onset bowel cancer include the autosomal dominant conditions of Lynch Syndrome, Familial Adenomatous Polyposis and PTEN Hamartoma Tumor Syndrome, as well as the autosomal recessive condition of MUTYH-Associated Polyposis. There is also an increased risk of early onset bowel cancer and other malignancies in individuals with biallelic mutations (homozygous or compound heterozygous) in the mismatch repair (MMR) genes. Screening recommendations are usually given to family members on the basis of the clinical diagnosis assisted by germline genetic testing for the proband and at-risk relatives.

For an individual with very early onset bowel cancer, careful consideration of the pathology and wider attention to phenotypic features and/or their family history provide clues as to whether there may be an underlying genetic predisposition. These include: age at diagnosis; clinical examination; presence or absence of polyps; results of immunohistochemistry (IHC) for the MMR gene proteins +/- BRAF testing; tumour microsatellite instability (MSI) testing, and family history of bowel cancer or other syndrome related cancers.

Sometimes, no clear conclusions can be reached about an underlying inherited mechanism, if any.

We will present the case of BK, who was diagnosed with caecal cancer at the age of 17. A right hemi-colectomy demonstrated a moderately to poorly differentiated adenocarcinoma, with a 40% mucinous component and tumour infiltrating lymphocytes. No precursor lesion was identified, and no polyps were identified in the distal bowel or in the resected bowel. Three lymph nodes out of 32 were positive for adenocarcinoma. Staging was negative for distant metastatic disease. Adjuvant chemotherapy with FOLFOX was given.

BK’s parents are second cousins. The only family history of cancer was a fifth-degree relative with bowel cancer. The results of genetic investigations will be presented, along with a reflection on the challenges for setting screening recommendations when two potentially at-risk relatives are severely intellectually delayed teenagers.

